# Characterization of murine CEACAM1 *in vivo* reveals low expression on CD8^+^ T cells and no tumor growth modulating activity by anti-CEACAM1 mAb CC1

**DOI:** 10.18632/oncotarget.26108

**Published:** 2018-10-02

**Authors:** Robbie L. McLeod, Minilik H. Angagaw, Toya Nath Baral, Liming Liu, Raymond Joseph Moniz, Jason Laskey, SuChun Hsieh, Mike Lee, Jin-Hwan Han, Hassan Issafras, Sarah Javaid, Andrey Loboda, Svetlana Sadekova, Joann A. O'Connor, Archie Tse, Juha Punnonen

**Affiliations:** ^1^ Merck & Co., Inc., Boston, MA, USA; ^2^ Merck & Co., Inc., Kenilworth, NJ, USA; ^3^ Merck & Co., Inc., Palo Alto, CA, USA

**Keywords:** CEACAM1, CC1, CD8+ T cells, syngeneic mouse model, pharmacokinetic

## Abstract

Carcinoembryonic antigen-related cell adhesion molecule 1 (CEACAM1) has been reported to mediate both tumorigenic and anti-tumor effects *in vivo*. Blockade of the CEACAM1 signaling pathway has recently been implicated as a novel mechanism for cancer immunotherapy. CC1, a mouse anti-CEACAM1 monoclonal antibody (mAb), has been widely used as a pharmacological tool in preclinical studies to inform on CEACAM1 pathway biology although limited data are available on its CEACAM1 blocking characteristics or pharmacodynamic-pharmacokinetic profiles. We sought to investigate CEACAM1 expression on mouse tumor and immune cells, characterize CC1 mAb binding, and evaluate CC1 in syngeneic mouse oncology models as a monotherapy and in combination with an anti-PD-1 mAb. CEACAM1 expression was observed at high levels on neutrophils, NK cells and myeloid-derived suppressor cells (MDSCs), while the expression on tumor-infiltrating CD8+ T cells was low. Unexpectedly, rather than blocking, CC1 facilitated binding of soluble CEACAM1 to CEACAM1 expressing cells. No anti-tumor effects were observed in CT26, MBT2 or A20 models when tested up to 30 mg/kg dose, a dose that was estimated to achieve >90% target engagement *in vivo*. Taken together, tumor infiltrating CD8+ T cells express low levels of CEACAM1 and CC1 Ab mediates no or minimal anti-tumor effects *in vivo*, as a monotherapy or in combination with anti-PD-1 treatment.

## INTRODUCTION

Carcinoembryonic antigen-related cell adhesion molecule 1 (CEACAM1), also known as CD66a or biliary glycoprotein-1, is a multifunctional transmembrane protein expressed in diverse cell types, including epithelial cells and certain cells of the immune system. CEACAM1, like other structurally related CEACAM 3-8 proteins, is a member of the Ig superfamily with a basic structure of sequentially ordered Ig-like domains. This protein serves as an adhesion molecule via homophilic and heterophilic interactions and participates in multiple physiological and pathophysiological cell to cell processes [1–4]. Moreover, direct immunomodulatory consequences have been suggested based on immune cell expression and the presence of ITIM motifs in the intracellular domain of the protein [5].

Early experiments investigating CEACAM1 in tumorigenesis denoted a pivotal role for CEACAM1 as a tumor suppressor. For example, prostate cancer cell line PC-3 transfected with CEACAM-1 demonstrated significantly lower growth rates and less tumorigenicity *in vivo* relative to controls [6]. Nittka *et al.* found that the absence of CEACAM1 on hyperplastic tumors correlated with reduced apoptosis of malignant cells [7]. Moreover, a lack of CEACAM1 in WAP-T tumor cells resulted in increased Wnt signaling, promoted cellular invasiveness, and strongly enhanced the rate of metastasis of mammary adenocarcinomas *in vivo* [8]. On the other hand, other investigations have suggested a role for CEACAM1 in angiogenesis and suppression of the neoantigen-specific anti-tumor response [3].

The expression of CEACAM1 on tumor cells has been implicated as a prognostic factor while different associations have been observed depending on tumor types and the stage of the cancer. Stable expression of CEACAM1 on melanoma cells enhanced invasion and migration of the cells *in vitro* [9] Reduced expression of CEACAM1 was reported upon malignant transformation in mice [10] and in samples of human colorectal cancer [11]. Similarly, CEACAM1 expression inversely correlated with survival of breast cancer patients [8]. Frequent loss of CEACAM1 in benign and malignant colorectal neoplasias was also reported [7]. Additionally, Thom *et al.*, found a significant positive correlation between CEACAM-1 expression on primary tumor lymph nodes and metastases [12]. Furthermore, expression of CEACAM1 on primary cutaneous melanoma lesions was linked to the development of metastatic disease and poor prognosis [13]. Thus, the biology of CEACAM1 appears complex, with disparate pathophysiological effects manifested in a context-dependent manner.

There has been an increasing interest by the oncology community to explore CEACAM1 as a potential target for cancer immunotherapy given its expression on tumor and immune cells and its potential immunomodulatory properties. It has been shown that CEACAM1 recruits SHP-1 to the TCR/CD3 complex resulting in reduced phosphorylation of CD3-zeta and ZAP-70 and consequently decreased activation of the ZAP-70 pathway *in vitro* [5]. CEACAM1 was also shown to modulate T cell activation and Th1 differentiation *in vivo*, while both activating [14, 15] and inhibitory [16, 17] effects have been reported. In addition to regulating the function of T cells, CEACAM1 acts as a critical survival factor for B cells [18] and modulates the cytolytic function of NK cells [19]. The majority of CD8+ T cells in human melanoma samples were reported to be CEACAM1+ [20] and CEACAM1 and TIM-3 are co-expressed on exhausted murine T cells during induction of tolerance [21]. Tumors implanted in CEACAM1 deficient mice exhibited impaired growth rate and mouse anti-CEACAM1 Ab CC1 prevented tumor growth in combination with anti-PD-L1 or anti-TIM-3 mAbs [21]. These data have led to a proposal that CEACAM1 plays a role as an immune checkpoint, similar to PD1, blocking productive anti-tumor responses *in vivo* [22].

In light of conflicting and partially opposing observations regarding the role of CEACAM1 in anti-tumor responses, we endeavored to further characterize CEACAM1 expression combined with evaluation of its potential tumor growth modulating properties using an anti CEACAM mAb. Anti-mouse CEACAM1 Ab CC1 has been widely used in preclinical studies to evaluate CEACAM1 biology *in vitro* and *in vivo* and several of the studies demonstrating immunomodulatory properties of CEACAM1 *in vivo* have been performed using CC1 [17, 19, 21, 23]. The mAb was initially generated by immunizing SJL/J mice with purified BALB/c intestinal brush border membranes and subsequently purified from a hybridoma derived by fusion of SP20 cells with spleen cells from the immunized mice [17]. While detailed functional characterization of CC1 Ab has been carried out in multiple studies, little information is available on the binding characteristics *in vitro*. Moreover, no data have been reported regarding the pharmacokinetic properties of CC1 in tumor bearing cancer models. In the present study, we sought to characterize expression profile of CEACAM1 and the effects of CC1 Ab in syngeneic mouse tumor models *in vivo*.

## RESULTS

### CEACAM1 expression on freshly isolated tumor infiltrating immune cells

**Figure 1:**
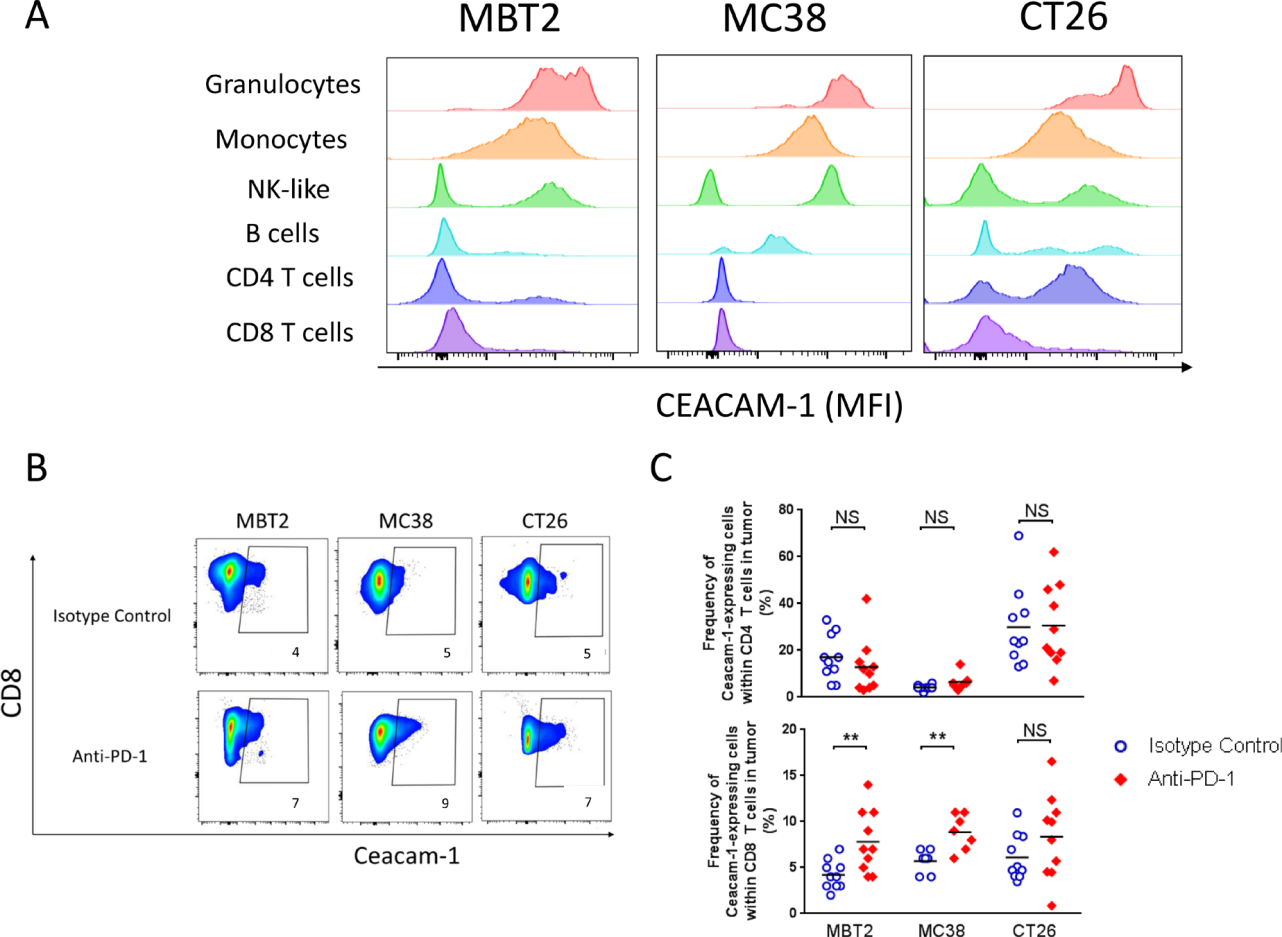
CEACAM1 expression on tumor-infiltrates within mouse syngeneic tumors in the presence or absence of anti-PD-1 (**A**) Basal level (with no *in vivo* treatment) of CEACAM1 expression on granulocytes (CD11b+ Ly6G+ Ly6Clow) and monocytic, monocytes (CD11b+ Ly6G−Ly6C+), NK-like (CD45+CD49b+), B cells (I/A-I/EhiCD11b-CD11c-CD45R+), CD4 (TCRβ+CD3+CD4+) or CD8 (TCRβ+CD3+CD8+) T cells within mouse syngeneic tumors, MBT2, MC38, or CT26, was measured by flow cytometry with anti-CEACAM1 antibody clone CC1. The histograms indicate the mean fluorescence intensity of CC1 staining. (**B** and **C**) The frequency of CEACAM1-expressing CD8 T cells in the indicated syngeneic tumors was measured at 4 days after the second dose (8 days after the initial dose) of either isotype control or anti-PD-1 injection. Examples of individual animals in each tumor are shown in (B) and the compiled frequencies of CEACAM1-expressing CD8 T cells are plotted in (C). T cells (TCRβ+CD3+CD4 or CD8+), Treg (FOXP3+HELIOS+ in the CD4+ T cell gate), G-MDSC (CD11b+I/A-I/ElowLy6G+Ly6Clow), M-MDSC (CD11b+ I/A-I/ElowLy6G-Ly6C+), cDC (I/A-I/EhighCD11blow/-CD11c+CD45R−), pDC (I/A-I/EhiCD11b-CD11clowCD45R+), macrophages (CD11b+ I/A-I/EhighLy6C+F4/80+), monocytes (CD11b+ I/A-I/EhighLy6C+F4/80low), B cells (I/A-I/EhiCD11b-CD11c-CD45R+), and “NK-like” cells (CD45+CD49b+).

To delineate a potential mechanism of CEACAM-1 blockade for anti-tumor therapy, the expression profile of surface CEACAM-1 in tumor infiltrates was comprehensively assessed using flow cytometry. Several mouse syngeneic tumor models, MBT2, MC38, CT26, and MB49, were used for the analyses to avoid a tumor type-specific bias. About 100 mm^3^ of subcutaneous tumors were established 7–10 days post implant. The CEACAM1 expression was first measured from untreated tumors by flow cytometry (Figure 1A). In all cases, high level of CEACAM1 expression was observed on granulocytic (CD11b+ Ly6G+ Ly6Clow) and monocytic (CD11b+ Ly6G− Ly6C+) myeloid cells from all tumors tested (Figure 1A, MB49 not shown). The frequency of CEACAM1-expressing CD4+ or CD8+ T cells and the expression level of CEACAM1 on these cells were low.

We next investigated the possibility that CEACAM1 on T cells could be up-regulated by PD-1 blockade upon TCR-mediated stimulation within the tumor microenvironment. Tumor-bearing mice were treated with anti-PD-1 mAb (muDX400) or its isotype control every four days for two doses. Four days after the second dose, the tumors were harvested and dissociated for immunophenotyping in order to assess a change in CEACAM1 expression (Figure 1B and 1C). The average proportion of CEACAM1+ cells remained less than 10% of total CD8 T cells and no statistically significant increase in frequency of CEACAM1+ CD8 T cells after two doses was observed. (Figure 1C, upper panel). The frequency of CEACAM1+ CD4 T cells appeared more variable in different tumors. For example, CT26 tumor has relatively higher frequency of CEACAM1+CD4 T cells compared to MBT2 or MC38. Nevertheless, the frequency of CEACAM1+ CD4 T cells remained unchanged after muDX400 *in vivo* treatment. Similarly, the frequency of CEACAM1+ populations in Treg and in all myeloid cells did not change before and after muDX400 *in vivo* treatment (data not shown).

### Effects of CC1 on ligand interactions of CEACAM1

**Figure 2:**
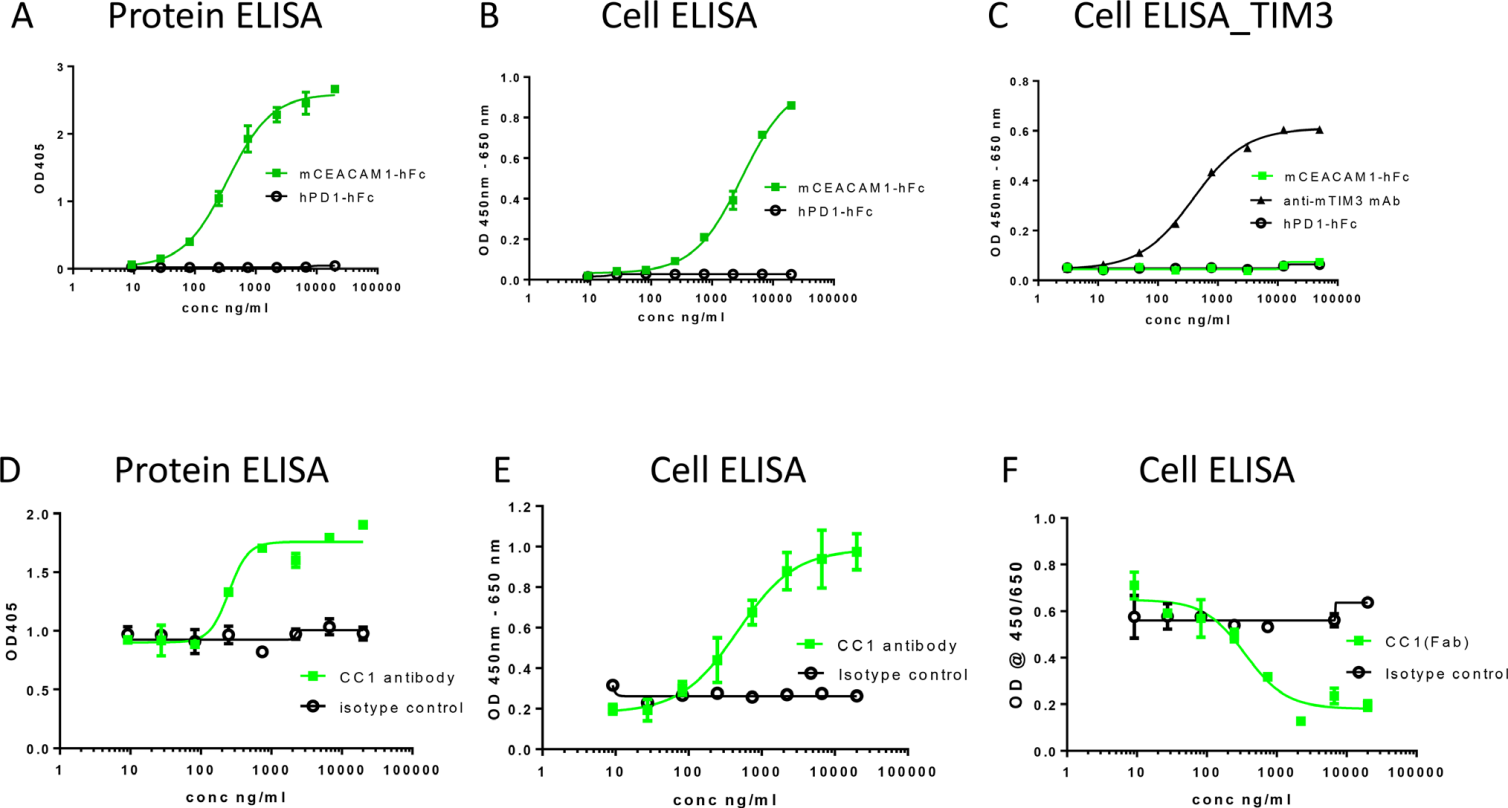
Ligand interactions of CEACAM1 and effects of anti-CEACAM1 Ab CC1 and its Fab fragment on homophilic CEACAM-1 interactions CEACAM1 ligand interactions were studied using protein- and cell-ELISAs by evaluating the binding of mCEACAM1-hFc (■) or control-hFc (PD1-Fc, ○) to (**A**) mCEACAM1-His protein, or (**B**) mCEACAM1-CHO cells. CEACAM1 interaction with mTIM-3 was evaluated using mTIM3-CHO cells (**C**) and anti-TIM-3 mAb was used as a positive control (▲). Effects of CC1 antibody antibody (■) or isotype control (○) on the homophilic interaction of mCEACAM1 were evaluated using (**D**) mCEACAM1-His in protein ELISA, or (**E**) mCEACAM1-CHO cells in cELISA. Effect of Fab fragment of CC1 (■) and isotype control (○) on mCEACAM1-hFc binding to mCEACAM1-CHO cells was studied in cELISA (**F**).

To evaluate the potential of CC1 to block its ligand interactions, we established protein- and cell-based ELISA assays. Mouse CEACAM1-CEACAM1 interaction was observed in both protein ELISA using recombinant CEACAM1 proteins and by cell ELISA (cELISA) using soluble protein and mouse CEACAM1-transfected cell lines (Figure 2A, 2B). CEACAM1 has also been reported to interact with TIM-3 [21] and therefore, we aimed to analyze this interaction by cELISA using recombinant mouse CEACAM1 protein and mouse TIM-3 transfected cell line. In this assay format, we did not observe any direct CEACAM1-TIM3 interaction, while expression of TIM-3 was confirmed using anti-TIM-3 mAb (Figure 2C). We also attempted to analyze the potential CEACAM1-TIM3 interaction by other methods such as Biacore and protein ELISA using human recombinant proteins, while no interaction between these two proteins was observed in these assay formats either (data not shown).

No blockade of CEACAM1-CEACAM1 interaction by CC1 was observed in either protein or cell-based ELISA (Figure 2D, 2E). In both assays we unexpectedly observed an increasing CEACAM1-CEACAM1 binding signal with increasing concentrations of CC1 (Figure 2D and 2E). Similar results were obtained by flow cytometry (data not shown) when soluble mCEACAM1 binding to mCEACAM1 transfected cells was analyzed in the presence of CC1 antibody. These data suggested that CC1 antibody may provide a bridging effect by linking two CEACAM1 molecules together rather than blocking the homophilic interaction of CEACAM1. For the bridging effect to occur, we hypothesized that a two-arm full-length IgG molecule would be needed. To address this question, we also generated a Fab fragment of CC1. When the Fab of CC1 was used in the same assay, we did not observe the increasing signal with increasing concentration of Fab. Instead, the Fab of CC1 blocked the CC1 homophilic interaction in cELISA (Figure 2F) and in protein ELISA (data not shown). Because of the undetectable binding of TIM-3 to CEACAM1 in our assays, the potential effect of CC1 on this interaction was not studied.

### Pharmacokinetic profile and target engagement of CC1 in mice

**Figure 3:**
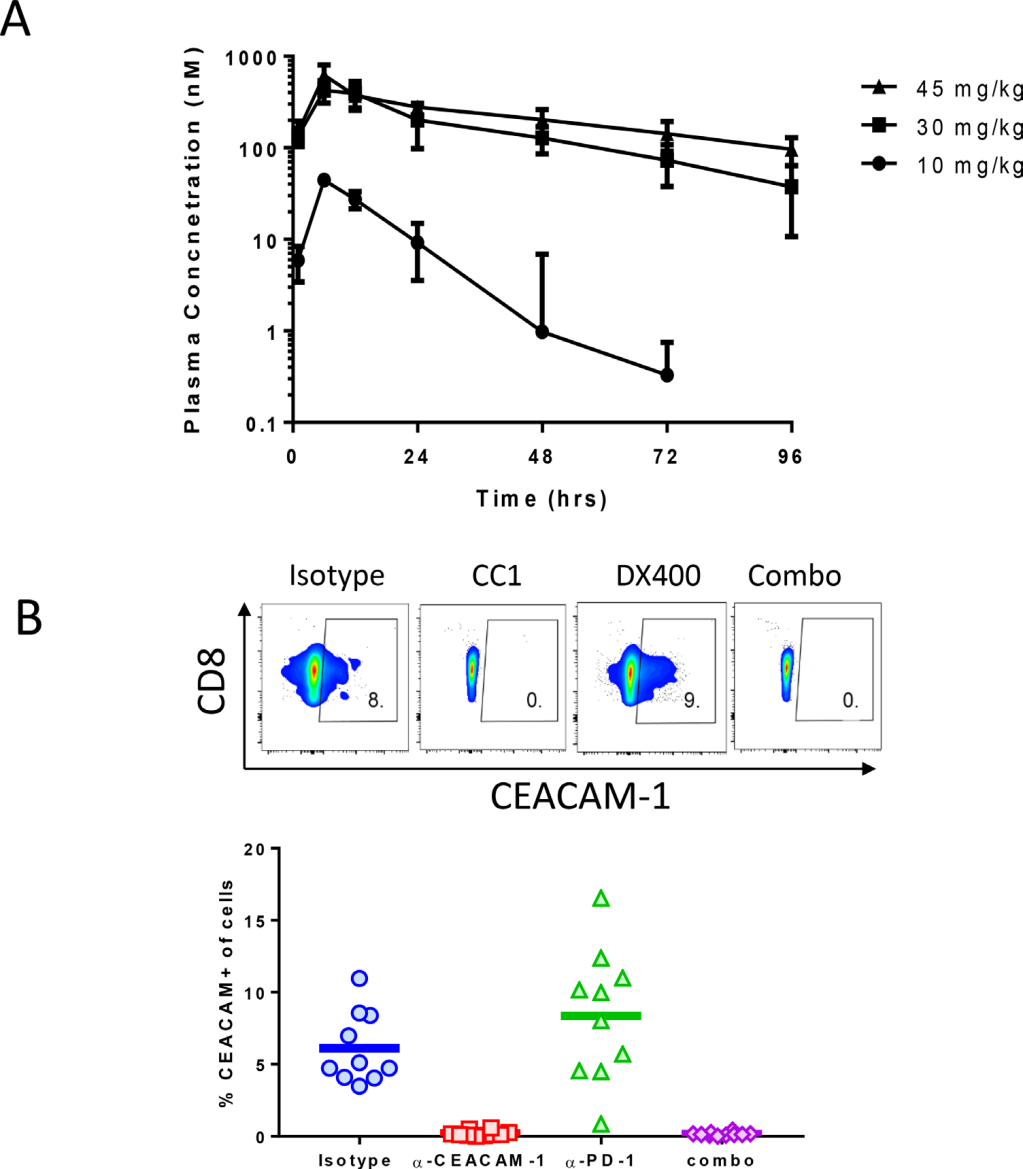
Concentration-time PK profile of CC1 in Balb/C mice Female BALB/C mice (8 weeks old, 4 mice/group) were dosed IP with CC1 at 10 mg/kg, 30 mg/kg or 45 mg/kg. Plasma from each mouse at hours 1, 6, 12, 24, 48, 72 and 96 following dosing was obtained by micro-sampling. Plasma CC1 concentration were determined with an ECL method (**A**). (**B**) panel displays the percent of CD8+ CEACAM cells after vehicle, CC1 (30 mg/kg), muDX400 (5 mg/kg) or CC1 plus muDX400 treatment.

The PK properties of CC1 were studied in Balb/c mice (Table 1). The PK parameters were non-linear between 10 mg/kg and 30 mg/kg with a significantly over-dose proportional increase for both CMax and AUC0-Inf and a significant difference in CL/F. At 10 mg/kg, CC1 exhibited fast clearance (CL/F 111 ± 34 mL/h/kg) with concentration dropping from ∼40 ug/mL at ∼6 h (CMax) to below 1 ug/mL 48 hr post dosing. This is an uncommon PK feature for an IgG1 mAb, suggesting that there is a significant sink effect mediated by target-associated drug disposition which could in part be due to an abundant expression of CEACAM1 on myeloid cells, including neutrophils [24]. At and above 30 mg/kg, the CC1 clearance was significantly reduced (CL/F 13 ± 5 mL/h/kg at 30 mg/kg and 11 ± 3 for 45 mg/kg), resembling a profile of non-targeted IgG1. Interestingly, above the 30 mg/kg dose, PK was linear because the CMax and AUC0-Inf were dose proportional between 30 mg/kg and 45 mg/kg. Consequently, the 30 mg/kg dose was chosen for further experiments in tumor models. Moreover, the simulated multi-dose (every 2 days) PK profile for the 30 mg/kg dose shows that the trough level of CC1 is approximately 313 nM, which is expected to sufficiently block CC1 binding *in vivo*. Indeed, full receptor occupancy of CEACAM-1 at a cellular level after *in vivo* CC1 treatments (30 mg/kg) was demonstrated by flow cytometry staining on CT26 tumor infiltrates using a fluorescence-labeled CC1. Both in the CC1 monotherapy group and the muDX400+CC1 combination therapy group, fluorescence-labeled CC1 was not able to stain the target receptor, CEACAM-1 (staining of CD8 T cells shown, (Figure 3B). This complete blocking was consistent in all mice in the CC1 monotherapy and muDX400+CC1 combination therapy groups (Figure 3B). These findings confirm that CC1 treatment has fully engaged CEACAM-1 on tumor infiltrates at a dose level of 10 mg/kg. We studied the impact of muDX400 at a dose level of 5 mg/kg Q5d as this has been used in many syngeneic mouse tumor models with good efficacy and exposure [25].

**Table 1:** Key Pharmacokinetic Parameters of CC1

PK parameters	10 mg/kg	30 mg/kg	45 mg/kg
C_max_ (nmol/mL)	44 ± 6	436 ± 132	622 ± 176
T_max_ (h)	6 ± 0	7.5 ± 3	6 ± 0
AUC_INF_ (h^*^nmol/mL)	641 ± 182	17525 ± 7218	27842 ± 7138
V_z__F (mL/kg)	1416 ± 610	442 ± 67	684 ± 126
Cl_F (mL/h/kg)	111 ± 34	13 ± 5	11 ± 3
t_1/2_ (h)	9 ± 1	26 ± 9	42 ± 4

Female BALB/c mice (8 weeks old, 4 mice/group) were dosed intraperitoneally (IP) with CC1 at 10 mg/kg, 30 mg/kg or 45 mg/kg. Plasma from each mouse at hours 1, 6, 12, 24, 48, 72 and 96 following dosing were obtained by micro-sampling. Plasma CC1 concentration were determined with an ECL method. PK Parameters were obtained with non-compartmental methods with Phoenix^®^ 32 WinNonlin^®^ 6.3 software.

### Antitumor activity of CC1 in syngeneic mouse studies

**Figure 4:**
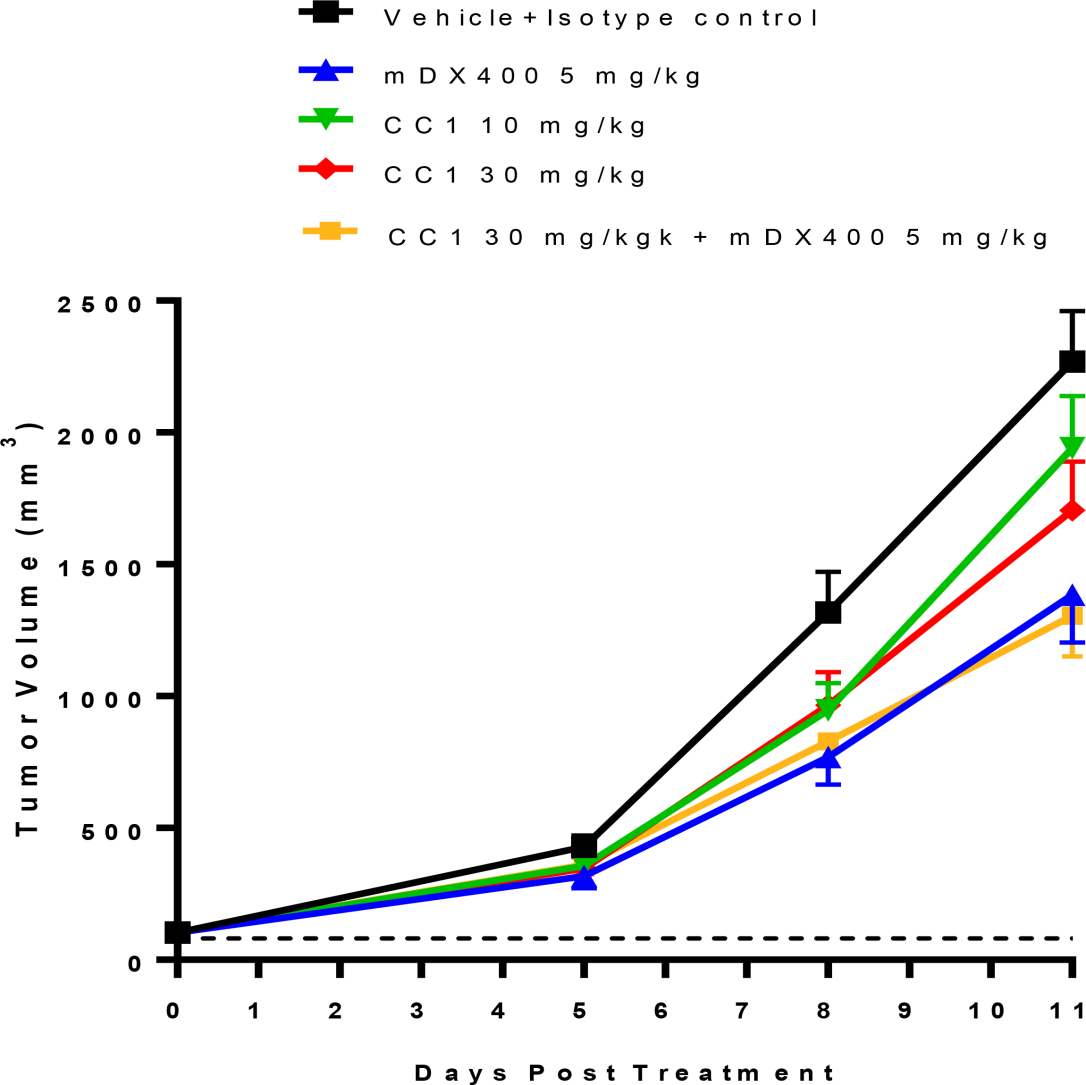
Evaluation of anti-tumor efficacy of CC1 alone and in combination with the anti-PD-1 antibody (muDX400) in a subcutaneous CT26 mouse syngeneic colon adenocarcinoma model CT26 tumor-bearing mice (mean tumor volume ∼100 mm^3^) were randomized into 5 treatment groups of 16 mice per group: (1) isotype control + vehicle control; (2) muDX400 (5 mg) + vehicle control; (3) isotype control + CC1 (10 mg/kg); (4) isotype control + CC1 (30 mg/kg) and (5) muDX400 (5 mg/kg) +CC1 (30 mg/kg). Significant anti-tumor activity was observed in the muDX400 (5 mg/kg) and muDX400 plus CC1 (30 mg/kg) combination arms as compared to controls. A significant difference was not observed between CC1 (10 and 30 mg/kg) treatment and control treatment. *P* < 0.05 (Kruskal–Wallis in conjunction with Mann–Whitney U post hoc analysis) compared to control at day 11.

**Figure 5:**
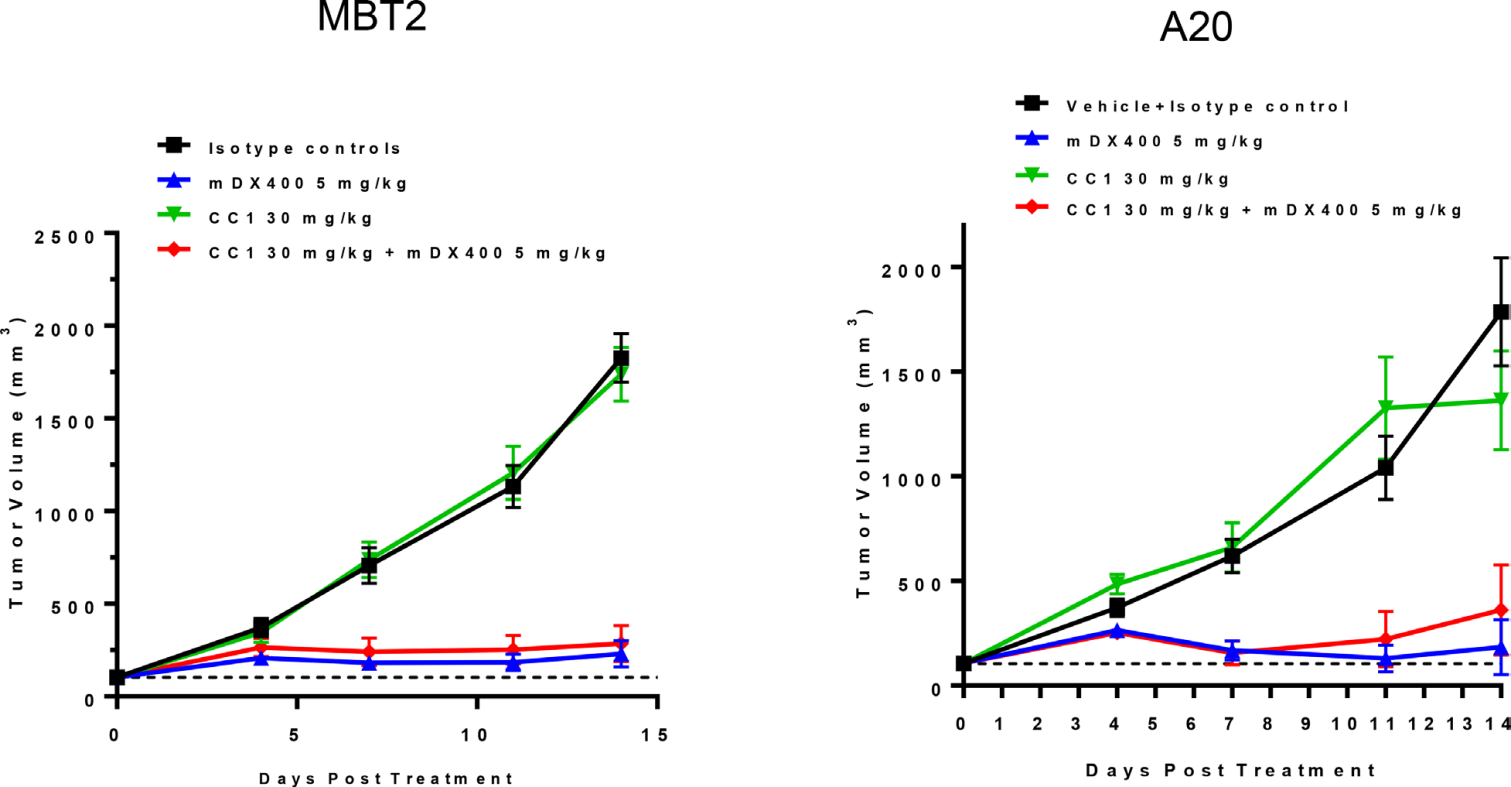
The effect of CC1 in alone and combination with the anti-PD-1 antibody (muDX400) in subcutaneous MBT2 (bladder) and A20 (B cell lymphoma) syngeneic mouse models CC1 (30 mg/kg) alone did not alter tumor growth rates compared to controls. muDX400 (5 mg/kg) and muDX400 plus CC1 (30 mg/kg) significantly slowed tumor growth. *P* < 0.05 (Kruskal–Wallis in conjunction with Mann–Whitney *U* post hoc analysis) compared to control at day 14.

To profile the impact of CC1 alone as a single agent and study the potential combinational effect with an anti-PD-1 antibody (muDX400), we utilized three subcutaneous mouse tumor models, CT26 (colorectal), MBT2 (bladder) and A20 (B cell lymphoma). CC1 (10–30 mg/kg) treatment with and without muDX400 (5 mg/kg) was well tolerated with no effects on mouse body weights across models and throughout the duration of each study (data not shown). In the CT26 experiments baseline tumor volumes for the control group at randomization were 100 mm^3^ and were not different from the treatment groups. By day 11, CT26 tumors in the control group increased approximately 20-fold. Figure 4 shows that CC1 (10 and 30 mg/kg, i.p.) administered every other day did not significantly attenuate tumor growth relative to controls. In contrast, muDX400 treatment (5 mg/kg, i.p. given every 4 days) inhibited tumor volume on day 11 by approximately 40–50%. There was no modulation of the muDX400 growth curve by co-administration of CC1 (30 mg/kg) indicating no observable combination benefit in this model. Similarly, no obvious combination activity with muDX400 and CC1 were demonstrated in either the MBT2 or A20 model (Figure 5).

**Figure 6:**
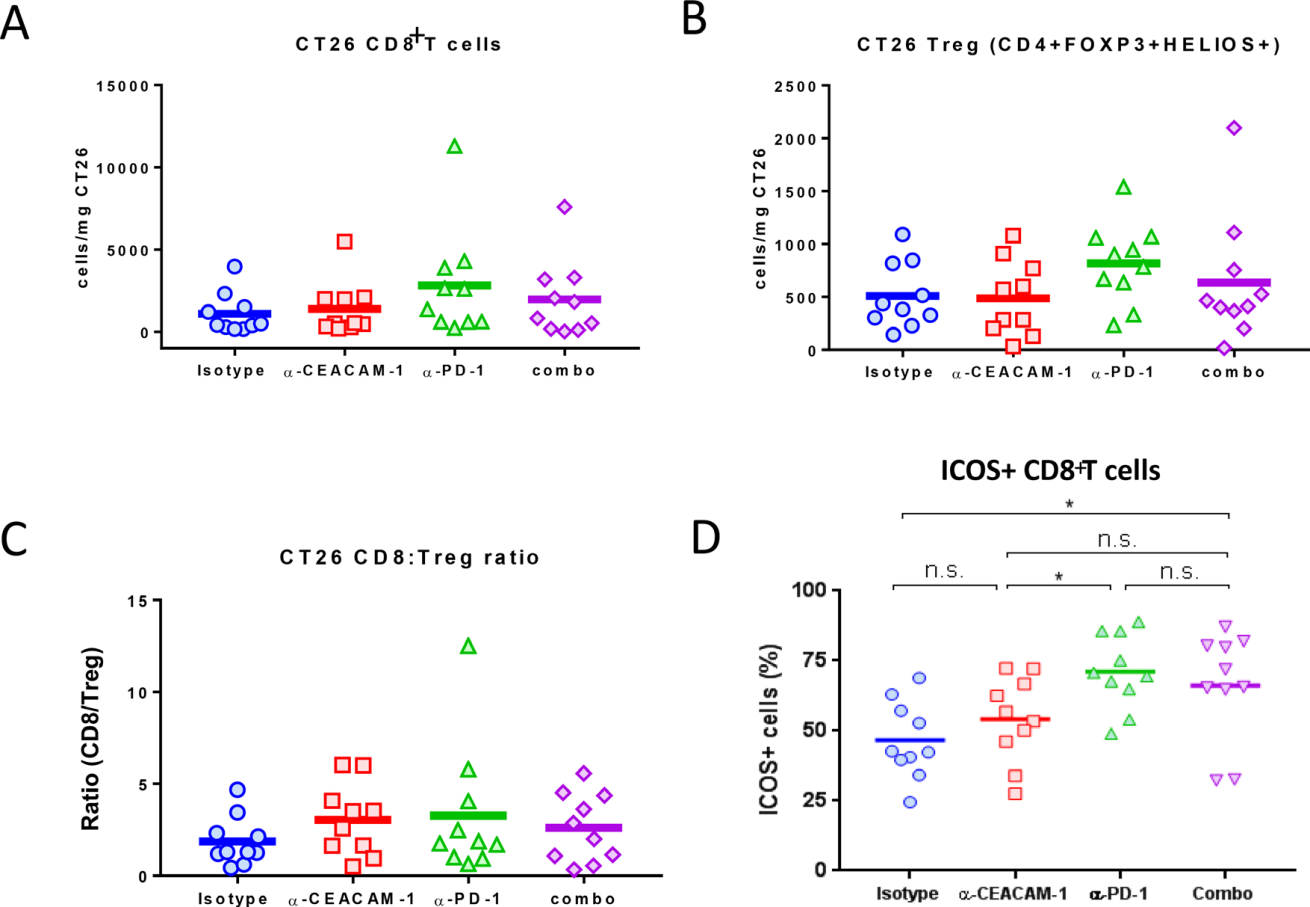
Quantitative and qualitative analyses of tumor-infiltrating T cells after anti-CEACAM monotherapy or its combination with anti-PD-1 treatment The numbers of total (**A**) CD8+ T cells or regulatory T cells (Tregs) (**B**) per milligrams of tumors have been determined at 4 days after the second dose (8 days after the initial dose) of either isotype control, anti-CEACAM or/and anti-PD-1 injection in CT26-bearing mice. (**C**) The ratios of absolute numbers of CD8+ T cells over those of Tregs (CD8/Treg) have been determined after anti-CEACAM monotherapy or its combination with anti-PD-1 treatment. (**D**) Frequencies of ICOS-expressing CD8+ T cells out of total CD8+ T cells in CT26 tumor have been determined by flow cytometry after anti-CEACAM monotherapy or its combination with anti-PD-1 treatment. The *P* values were obtained by Mann–Whitney test. ^*^P < 0.05.

To assess whether intratumoral immune activation is altered following monotherapy or combination treatment with CC1 and muDX400, tumor infiltrates were isolated from the CT26 model. Multiple parameters were analyzed: absolute numbers of CD8+ and Treg cells, ratio between CD8+ and Treg cells, and the frequency of ICOS+ CD8+ T cells in CT26 tumor at day 8 post initial dosing of each group. No significant increase in numbers of CD8+ T cells or Treg cells were found in the tumors of mice treated with muDX400, CC1 or the combination (Figure 6A and 6B). There was no effect of treatment on CD8+ T cells/Treg ratios (Figure 6C). In previous studies, it has been demonstrated that one of the co-stimulatory receptors, inducible co-stimulator (ICOS) is expressed on CD8+ T cells when an immune check point blockade is effective [26]. For a qualitative assessment of intratumoral CD8 T cells after each treatment, the frequency of ICOS-expressing CD8 T cells was monitored. muDX400 monotherapy group showed a significant expansion of ICOS+ CD8 T cells in CT26 tumors, compared to those in the isotype control group. CC1 did not significantly increase ICOS expression as a monotherapy or when combined with muDX400 (Figure 6D). In addition, no consistent changes in the proportions of CD4+, NK cells, B cells, monocytes, dendritic cells or myeloid-derived suppressor cells (myeloid or granulocytic) upon CC1 treatment were observed (data not shown).

## DISCUSSION

CEACAM1 is a pleiotropic cell surface molecule expressed on diverse cell types. Its expression levels are rapidly modulated upon activation, adding to the complexity of defining an unambiguous role of CEACAM1 in health and disease. In the current study, we (1) evaluated the potential of CC1 to block its ligand interactions, (2) studied CEACAM1 expression on TILs from syngeneic tumor models (3) defined the PK characteristics of CC1 after IP administration and (4) profiled the anti-tumor effects of CC1 in syngeneic mouse models.

We found that CEACAM1 was expressed at relatively high levels on freshly harvested tumor infiltrating B, NK cells, and MDSCs, although the proportion of these cells was low. Additionally, the present results demonstrated low expression of CEACAM1 on CD8+ tumor infiltrating T cells across three different tumor models. Our findings diverge from previously published observations in which CEACAM1 was identified as a marker of exhausted T cells in the tumor microenvironment with majority of the CD8+ T cells in human melanoma samples expressing CEACAM1 by IHC [20]. In mice, a significant fraction of tumor infiltrating CD8+ T cells have also been reported to express CEACAM1 [21]. The reason for apparent differences in tumor CEACAM1 expression profiles between our study and others is unclear. Differences in the tumor microenvironments or staining conditions cannot be ruled out, but this appears unlikely due to strong staining of CC1 observed on myeloid cells and CT26 tumor cells used by both us and others [21]. Immune checkpoint inhibitors, such as anti-PD-1 mAbs, have demonstrated remarkable clinical benefits in multiple cancer types [27–29]. Given that CEACAM1 expression on both immune cells and tumor cells is reported to be upregulated following activation by IL-2 or tumor cell contact [30, 31], we also investigated whether anti-PD-1 treatment enhances CEACAM1 expression on CD8+ T cells. CEACAM1 expression on tumor infiltrating T cells remained low whether or not the animals were treated with anti-PD-1 (muDX400) over multiple doses at levels that provided strong anti-tumor activity *in vivo*. These data suggest that CEACAM1 has a low impact as a direct regulator of tumor infiltrating T cells.

The anti-mouse CEACAM1 Ab, CC1, has been used in a number of preclinical studies to evaluate CEACAM1 as a potential anti-tumor target [17, 19, 21, 23], however we were not able to find any published studies describing the ligand blocking capacity of this antibody. We therefore established both protein- and cell-based ELISA assays to evaluate the impact of CC1 on homophilic CEACAM1-CEACAM1 interactions. Unexpectedly, inclusion of the CC1 mAb in these assays enhanced the signal resulting from CEACAM1 interactions, suggesting that CC1 enhances rather than blocks, CEACAM1-CEACAM1 interactions. Our data also appear to indicate that the CC1 Ab is able to bind to two CEACAM1 molecules simultaneously consequently establishing a bridging CEACAM1-CC1-CEACAM1 relationship. For the bridging effect to occur, it appears that a two-arm full-length IgG molecule is needed. This conclusion is supported by our results showing that the Fab fragment of CC1 did not increase signaling in the homophilic CEACAM-1CEACAM1 binding assays. Additional studies are needed to fully evaluate the functional significance of such a CEACAM1-CC1-CEACAM1 binding profile.

CEACAM1 has also been reported to interact with TIM-3 [21] and, thus, we studied the potential effects of CC1 on this interaction. However, we were unable to reproducibly detect CEACAM1-Ig or CEACAM1-His binding of TIM-3 and hence, the evaluation of CC1 in these assays was not feasible. Minimal binding of TIM-3 to CEACAM1-Ig or CEACAM1-His was observed in both cell-based assays using mouse reagents (Figure 2C) and protein-based assays using human reagents (data not shown). Since CEACAM1–TIM-3 interactions were shown to occur both in the *cis* (through the membrane distal N-terminal domains of the molecules) and *trans* (through their N-terminal domains), the development of additional assays to assess potential effects of CC1 on CEACAM1–TIM-3 interactions will be needed. The current data do not reveal the reason for the lack of detectable binding in our assays, while low affinity interactions are likely to play a role. In addition, as galectin-9 has been identified as another ligand for TIM-3 and is ubiquitously expressed in multiple tissues and cell types [32], potential interference by galectin-9 in the cell-based assays cannot be ruled out. However, it should be noted that no prior studies have suggested modulation of CEACAM1-Tim-3 interactions by the CC1 Ab.

Previous studies have provided functional evidence for tumor growth modulating activities of CEACAM1. Reduced growth of CT26 tumor cells was observed in CEACAM1 deficient mice [21]. In addition, treatment with anti-CEACAM1 Ab CC1 in combination with TIM-3 or PD-L1 blockade resulted in robust CT26 tumor growth inhibition and an increase in tumor infiltrating CD8+ T cells in the CT26 model [21]. One limitation of these prior studies is that they did not report the pharmacokinetic characteristics or the level of CEACAM1 target engagement achieved in these experiments. Our experiments found that CC1 has a relative short T1/2 in mice and associated with a rapid clearance, which in part may be related to high CEACAM1 expression on myeloid cells facilitating receptor-mediated deposition. In our *in vivo* syngeneic tumor models, CT26, MBT2 and A20, anti-CEACAM1 Ab CC1 administered as a single agent therapy or in combination with an anti-PD-1 mAb (muDX400), provided no tumor growth inhibition. It is important to point out that full target engagement was achieved in these *in vivo* experiments, based on our tumor immunophenotyping experiments showing greater than 90% suppression of cell surface CEACAM1 staining in CC1-treated animals and the fact that we maintained a plasma trough levels well above the receptor binding IC50 (at a dose of 30 mg/kg).

We next studied the possibility that the CC1 Ab modulated the phenotypes of tumor infiltrating immune cells. Flow cytometry analysis of tumor-infiltrating cells freshly isolated from syngeneic tumors revealed no changes in the proportions of CD4+, CD8+ T cells, Treg cells, NK cells, B cells, monocytes, dendritic cells or myeloid-derived suppressor cells (myeloid or granulocytic) upon CC1 treatment. In addition, no phenotypic changes were observed in tumor infiltrating T cells derived from mice treated with CC1 as a monotherapy or in combination with the anti-PD-1 Ab.

Taken together, the present study demonstrates low expression of CEACAM1 on tumor infiltrating mouse T cells, while significant expression was observed on B cells, NK cells and MDSCs. This expression profile is similar to that observed in freshly isolated human tumors (Lee *et al.*, manuscript in preparation/submitted). Evaluation of the potential role of CEACAM1 in anti-tumor responses in syngeneic tumor models using the anti-CEACAM1 mAb CC1 did not reveal any anti-tumor benefits of the mAb, as a monotherapy or in combination with an anti-PD-1 mAb. However, while the CC1 Ab has been widely used in prior *in vivo* studies, our data suggest that the mAb facilitates, rather than blocks, CEACAM1-CEACAM1 interactions. Therefore, further studies using blocking Abs will be required to thoroughly understand the role CEACAM1 in tumorigenesis and anti-tumor responses *in vivo*. The potential role of CEACAM1 on B cells, NK cells and MDSCs was not addressed in this study and will be the focus of future studies. Low or minimal expression of CEACAM1 on tumor infiltrating T cells suggests that the primary function of CEACAM1 *in vivo* is mediated via cells other than CD8+ T cells, and the potential role of CEACAM1 in immuno-oncology remains to be established.

## MATERIALS AND METHODS

### ELISA and cell ELISA

Evaluation of the homophilic interaction of CEACAM1 or interaction of CEACAM1 to TIM-3 was performed by protein ELISA or cell ELISA (cELISA). For cELISA, microtiter plates were seeded with mCEACAM1 or mTIM-3 transfected CHO cells 1 or 2 days prior so that on the assay day the cells were ∼80% confluent. On the assay day, the cell culture supernatant was aspirated and 50 µl of serially diluted mCEACAM-hFc (Sino Biologics) or control protein with hFc were added to the microtiter plates. Dilution was done in cell culture medium (DMEM (Gibco) supplemented with 10% fetal bovine serum. After 30 minutes of incubation at room temperature, plates were washed three times with washing buffer (PBS containing 0.05% Tween 20). Afterwards, 50 µl of horseradish peroxidase-conjugated goat anti-human IgG (Jackson ImmunoResearch) diluted 1:2000 in cell culture medium was added, and incubated at room temperature for 30 min. Finally, plates were washed five times, and 50 µl of 1-Step Nitro TMB-ELISA substrate (ThermoFischer) per well was added for 5 min. Reactions were stopped by adding 50 µl of TMB Stop solution (KPL), and the absorbance was measured at 450–620 nm. Protein ELISA is performed very similar to cELISA with few difference. For protein ELISA, microtiter plates were coated with 50 µl of mCEACAM-His (Sino Biologics) 1 µg/ml in PBS at 4° C over-night. Next day, plates are washed three times with wash buffer and blocked with 200 µl Supeblock (Thermo Scientific) for one hour at room temperature. After washing the plates, addition of mCEACAM1-hFc or control hFc, addition of anti-human HRP and incubation steps were same as in cELISA. Finally, plates were washed five times, and 50 µl of ABTS peroxidase substrate (KPL) per well was added. After 5 min. the absorbance was measured at 405 nm.

For the evaluation of the effect of CC1 antibody on homophilic CEACAM1 interaction, the ELISA and cELISA assays used were similar as described above with some modifications. For ELISA, coating and blocking were performed the same way as above. Afterwards, 50 µl of a 1:3 serially diluted solution of CC1 antibody (starting at 20 µg/ml) or isotype control were added to the microtiter plates. After a 30 min incubation at room temperature, 50 ul of mCEACAM-hFc (Sino Biologics) at 1 µg/ ml was added and incubated for another 30 mins. Next, plates were washed three times with washing buffer as above and bound mCEACAM1-hFc was detected using a horseradish peroxidase-conjugated goat anti-human IgG (Jackson ImmunoResearch) as described above. For cELISA, a similar protocol was used except that mCEACAM1-hFc was used at 10 µg/ml and all other steps were performed the same way as for the ELISA assay.

### Animal care and use

All *in vivo* studies were performed in accordance to the guidelines of the Institute for Laboratory Animal Research (ILAR). These studies were part of an institutional animal care and use committee (IACUC)-approved protocol and animals were housed in an AAALAC International accredited research facility. Since all experimental key readouts were terminal, all study animal groups were used once only and were euthanized at the end of each study.

### Pharmacokinetic profile of CC1 in mice

Female BALB/c mice (8 weeks old, 4 mice/group) were dosed intraperitoneally (IP) with CC1 at 10 mg/kg, 30 mg/kg or 45 mg/kg. A previous described serial micro-sampling method was used to obtain plasma from each mouse at hours 1, 6, 12, 24, 48, 72 and 96 following dosing [33]. Plasma CC1 concentration were determined with an electrochemiluminescence (ECL) method. Briefly, recombinant mouse CEACAM-1 (SinoBiological) was used as a capture reagent and sulfoTAG AffiniPure goat anti-mouse IgG was used as a detection reagent. Twenty-five microliter of the capture reagent was added to each well of MA6000 96 Small Spot plate (Meso Scale Discovery) and incubated overnight at 4° C with shaking. After washing with 0.05%Tween20 in PBS three times, the plate was blocked with 5% bovine serum albumin BSA at 150 µL per well and incubated at room temperature for 1 hour with shaking. After additional washings, Twenty-five µL of calibration standard, quality control or sample was added to each well of the washed plate and incubated at room temperature for 1 hour with shaking. After washes, the plate was incubated with 0.5 µg/mL of sulfoTAG AffiniPure goat anti-mouse IgG for 1 hour at room temperature. Then the plate was washed three times and 150 µL of 1× Reading Buffer T (Meso Scale Discovery) was added to each well of the plate followed by reading on a Meso Sector s600 Model 1201 (Meso Scale Discovery). CC1 concentration data were analyzed and key PK parameters calculated using non-compartmental methods with Phoenix^®^ 32 WinNonlin^®^ 6.3 software. Data were also analyzed with a WinNonlin^®^ 2 compartmental model which was used to simulate multi-dose PK profiles.

### Tumor cell culture

CT26 tumor cells were cloned from N-nitroso-N-methylurethane (NNMU)-induced, undifferentiated colon carcinoma cell line. MBT-2 was initiated from primary N-[4-(5-nitro-2-furyl)-2-thiazolyl] formamide (FANFT) induced murine bladder tumors arising in C3H/He mice. CT26 and MBT-2 cells were cultured in RPMI-1640 and DMEM medium respectively containing 10% fetal bovine serum. Both mouse colon adenocarcinoma cell-line, MC38, and a mouse bladder carcinoma cell-line, MB49 were cultured in DMEM medium with 10% fetal bovine serum. The tumor cells were then grown in tissue culture flasks in a humidified incubator at 37°C at atmosphere conditions of 5% CO2 and 95% air. A20 cell line is a BALB/c B cell lymphoma line derived from a spontaneous reticulum cell neoplasm. The cells were cultured in RPMI-1640 medium containing 10% fetal bovine serum and 0.05 mM 2-mercaptoethanol in tissue culture flasks as described above.

### Activity of CC1 in syngeneic mouse models

Eight week old female BALB/c and C3H mice were purchased from Taconic (New York, NY, USA). Tumor efficacy studies were conducted in three different syngeneic mouse models, namely colon carcinoma (CT26), bladder (MBT2) and B cell lymphoma (A20). For CT26 and A20 models, BALB/c mice were inoculated subcutaneously into the right lower flank with 1 × 10^6^ cells. For MBT2 experiments, C3H mice were injected with 1 × 10^6^ cells (lower right flank). Tumors were measured with calibers two times per week and tumor volumes determined using the relationship: (volume (mm^3^) = length × width^2^/2). Mice were euthanized when tumors reached a volume of 2000 mm^3^. Randomization of mice to treatment groups occurred when mean tumor volumes reached approximately 100 mm^3^. We examined increasing dose levels of CC1 (10 and 30 mg/kg; IP, QD every other day) on tumor growth in the CT26 model. Additionally we studied the effect of anti-CEACAM1 treatment alone (CC1 30 mg/kg; IP, QD every two days) and in combination with muDX400 (5 mg/kg; which is a murinized version of surrogate anti-mouse PD1 mAb.; QD every 5 days) in the CT26, MBT2 and A20 models. Vehicle controls received mouse IgG1 antibody and vehicle. The dose of muDX400 was chosen based on demonstrated efficacies achieved in syngeneic mouse tumor models [25].

### Flow cytometry

Immunophenotyping of tumor cells from the CT26, MC38, MB49, and MBT2 models conducted by flow cytometry. Tumor cells were excised at the indicated time point, minced by mechanical digestion, and subjected to enzymatic digestion using a gentleMACS and the mouse tumor digestion kit per the manufacturer’s instructions (Miltenyi Biotec). Following digestion, single cell suspensions were obtained after filtration and multiple washes, from which the absolute live cell count was determined using a ChemoMetec NucleoCounter. Cells suspension were then stained with a fixable LIVE/DEAD stain (Life Technologies), washed, and exposed to mouse Fc-Blok (BD Biosciences). Immune cell profiling was conducted using anti-mouse antibodies (clone) purchased from either BD Biosciences, BioLegend, or eBioscience/Thermo: CD45 (30-F11); CD8 (53–6.7); CD3 (145-2C11); TCRβ (h57597); CD4 (GK1.5); CD11b (M1/70); Ly6G (1A8); Ly6C (AL-21); CD45R (RA3-6B2); CD11c (N418); I-A/I-E (M5/114.15.2); or CD66a (clone CC1). For intracellular staining, cells were permeabilized using Foxp3/Transcription Factor Staining Buffer Set and incubated with anti-mouse Foxp3 (FJK-16S) and HELIOS (22F6) (eBioscience). Stained cells were fixed in 4% formaldehyde, washed with PBS, and stored at 4° C until analysis on a BD LSRFortessa (BD Bioscience). Data analysis was performed using FCS Express (De Novo Software) or FlowJo (FlowJo LLC). 10 mice per treatment group were included in all flow cytometry analyses. Immune subset discrimination was determined by gating on live, CD45+ cells in the leukocyte gate (FSC vs SSC size discrimination) via the following gating schemes; T cells (TCRβ+CD3+CD4 or CD8+), Treg (FOXP3+HELIOS+ in the CD4+ T cell gate), G-MDSC (CD11b+I/A-I/ElowLy6G+Ly6Clow), M-MDSC (CD11b+ I/A-I/ElowLy6G-Ly6C+), cDC (I/A-I/EhighCD11blow/-CD11c+CD45R−), pDC (I/A-I/EhiCD11b-CD11clowCD45R+), macrophages (CD11b+ I/A-I/EhighLy6C+F4/80+), monocytes (CD11b+ I/A-I/EhighLy6C+F4/80low), B cells (I/A-I/EhiCD11b-CD11c-CD45R+), and “NK-like” cells (CD45+CD49b+). For each population, a minimum of 250 events were acquired.
